# Optimizing genome editing efficiency in *Streptomyces fradiae* via a CRISPR/Cas9n-mediated editing system

**DOI:** 10.1128/aem.01953-24

**Published:** 2025-01-22

**Authors:** Yuhan Wu, Hui Jin, Qiang Yu, Zihan Wei, Jiang Zhu, Xiangqi Qiu, Gan Luo, Junhui Li, Yangyang Zhan, Dongbo Cai, Shouwen Chen

**Affiliations:** 1 State Key Laboratory of Biocatalysis and Enzyme Engineering, Environmental Microbial Technology Center of Hubei Province, College of Life Sciences, Hubei University 644987 , Wuhan, China; 2 Lifecome Biochemistry Co. Ltd., Nanping, China; Danmarks Tekniske Universitet The Novo Nordisk Foundation Center for Biosustainability, Kgs. Lyngby, Denmark

**Keywords:** *Streptomyces fradiae*, CRISPR/Cas9^D10A^, single strand breaks repair, metabolic engineering, neomycin

## Abstract

*Streptomyces fradiae* is an important bioresource to produce various antibacterial natural products, however, the time-consuming and labor-intensive genome editing toolkits hindered the construction and application of engineered strains, and this study aimed to establish an efficient CRISPR/Cas9n genome editing system in *S. fradiae*. Initially, the CRISPR/Cas9-mediated editing tool was employed to replace those awkward genome editing tools that relied on homologous recombination, while the off-target Cas9 exhibited high toxicity to *S. fradiae* Sf01. Therefore, the nickase mutation D10A, high-fidelity mutations including N497A, R661A, Q695A, and Q926A, and thiostrepton-induced promotor P*_tipA_* were incorporated into the Cas9 expression cassette, which reduced its toxicity. The deletion of single gene *neoI* and long fragment sequence (13.3 kb) were achieved with efficiencies of 77.8% and 44%, respectively. Additionally, the established tool was applied to facilitate the rapid deletion of *nagB*, replacement of P*_frr_* with P_ermE_*, and integration of exogenous *vgbS*, with respective efficiencies of 77.8%, 100%, and 67.8%, and all of the above modification strategies benefited neomycin synthesis in *S. fradiae*. Taken together, this research established an efficient CRISPR/Cas9n-mediated genome editing toolkit in *S. fradiae*, paving the way for developing high-performance neomycin-producing strains and facilitating the genetic modification of *Streptomyces*.

**IMPORTANCE:**

This study describes the development and application of a genome editing system mediated by CRISPR/Cas9n in *Streptomyces fradiae* for the first time, which overcomes the challenges associated with genome editing caused by high GC content (74.5%) coupling with complex genome structure, and reduces the negative impact of “off-target effect.” Our work not only provides a facile editing tool for constructing *S. fradiae* strains of high-yield neomycin but also offers the technical guidance for the design of a CRISPR/Cas9n mediated genome editing tool in those creatures with high GC content genomes.

## INTRODUCTION

*Streptomyces fradiae*, a member of the *Streptomyces* family, has been primarily utilized for the production of various antibiotics (1, 2), alkaloids (3), auxin (4), etc. However, the lack of a suitable genome editing tool has been a barrier to enhancing biochemical production in *S. fradiae* through metabolic engineering. The genome of *S. fradiae* demonstrates a spontaneous mutation rate exceeding 0.1%, and approximately 0.05% of spores exhibit chromosome deletions (5), without harmful effects on growth (6). Furthermore, *S. fradiae* possesses a GC content of 74.5% (NCBI ID: ASM870442v1), resulting in the formation of an intricate three-dimensional genetic structure and presenting significant obstacles for genome modification (7).

Recently, the construction of industrial *Streptomyces* strains has largely relied on editing vectors via homologous recombination, and a number of gene editing strategies have subsequently been derived, including PCR targeting (8–10), I-SceI editing (11, 12), and Cre-loxP editing systems (13). Nevertheless, the common limitation of the aforementioned methods is that they relied on the occasional crossovers in the division phase, which is subject to a great deal of contingency and uncertainty. In particular, given the long lifespan of *Streptomyces*, the process of subculturing and verifying each generation is time-consuming, and the potential for reverse mutation would result in an incalculable extension of processing time. Furthermore, PCR targeting systems do not allow for seamless editing, the I-SceI editing system has species differences in editing efficiency, and the Cre-loxP editing system needs repetitive preparation. Therefore, an alternative editing tool for *S. fradiae* genome modification was eagerly awaited.

CRISPR/Cas9, derived from the defensive system in *Streptococcus pyogenes* (14), has been extensively studied and used as a generalist gene editing tool. In comparison to the aforementioned genome editing methods via homologous recombination, the CRISPR/Cas9-mediated genome editing tool has the distinct advantage of achieving traceless editing and resistant marker deletion simultaneously, negating the necessity for an additional step (15), which enhances the efficiency of the experimental process and has also led to the development of CRISPR/Cas9 editing technology in *Streptomyces*. For instance, Cobb et al. developed the pCRISPomyces system for genome editing of *Streptomyces lividans*, which was shown to be capable of deleting large chromosomal fragments (up to 30 kb) with editing efficiencies from 70% to 100% (16). Tong et al. constructed the CRISPR-Cas9 and CRISPR-base editing systems in *Streptomyces coelicolor*, achieving double strands break (DSB) dependent genome editing and DSB-free genome editing, respectively (17, 18). Je et al. developed the CaExTun platform enabled rapid screening of a suitable promoter to Cas9, and was also employed to modify four model *Streptomyces* strains (19). The successful application of CRISPR technology in model *Streptomyces* prompted us to develop the same approach in *S. fradiae*, the non-model *Streptomyces*.

However, CRISPR/Cas9 has a tolerance for the mismatch between sgRNA and target DNA, which may result in non-specific cutting (20). This phenomenon is defined as an “off-target effect,” and is also considered a toxicity of Cas9. Meanwhile, high GC content in the genome might reduce the targeting specificity of CRISPR/Cas9 and increase the risk of “off-target effect.” Unfortunately, according to the NCBI genome database, *S. fradiae* (NCBI ID: ASM870442v1) is one of the *Streptomyces* with the highest GC content (74.5%), which is vulnerable to “off-target effect.” In light of the above considerations, Cas9n (Cas9 nickase) has been identified as an ideal substitute for Cas9, and the D10A mutated Cas9 only leads to single-strand breaks (21), which reduces the negative impact from the “off-target effect” (22).

Neomycin is an important antibiotic produced by *S. fradiae*, which is widely used in the fields of veterinary drugs, feed additives, etc. However, the low genetic transformation efficiency hinders metabolic engineering breeding for high-yield production of neomycin. In this study, we aimed to develop a CRISPR/Cas9n-mediated genome editing system in *S. fradiae*, including an editing vector and optimized conjugation conditions. It was expected not only to provide an efficient tool for targeted genome editing in *S. fradiae* but also to offer technical guidance for the design of CRISPR/Cas9-related editing tools in those high GC content creatures.

## RESULTS

### Optimization of conjugation conditions for the transfer from *Escherichia coli* to *S. fradiae*

*S. fradiae* Sf01 is an industrial strain used to produce neomycin, which was isolated and purified for many years, resulting in a more complex genome structure and background. The traditional genome editing plasmid pKC1139, which was based on homologous recombination, presented a significant challenge in terms of transfer into Sf01 by conjugation. The conjugation efficiency was observed to be less than 10⁻⁹. On account of this, the initial investigation tended to rectify the unsuccessful outcome by optimizing the conjugation conditions.

An appropriate solid medium was a crucial factor in the formation of strong strains, and the introduction of Mg^2+^ might enhance the conjugation efficiency (23). We first compared the capability of five solid media including SFM, IWL4, 2CMY, GS-1, and ISP2. In SFM solid medium, Sf01 exhibited optimal growth, with about 2 × 10^9^ spores (Fig. S1A). Subsequently, SFM was further optimized through the addition of a gradient-increasing concentration of MgCl_₂_ (0 to 60 mM), with the objective of identifying an appropriate MgCl_₂_ supplementation that could enhance the conjugation efficiency. pKC1139 was used as donor plasmids for the conjugation experiment, and about 1 × 10^8^ *S. fradiae* spores were used as recipients. When SFM contained 50 mM MgCl_2_, the conjugation efficiency of pKC1139 was the highest, which was 2.3 × 10^−7^ (23 conjugants) (Fig. S1B). SFM media containing 50 mM MgCl_2_ was called SFMM media.

On the basis of SFMM media, we also explored the influence of three important parameters on the conjugation efficiency, including the heat shock temperature (Fig. S1C), the antibiotic addition time (Fig. S1D), and the ratio of donor to recipient of conjugation (Fig. S1E). Optimal conditions were determined as 50°C of heat shock, 18 h of antibiotic addition time after conjugation, and 20:1 of donor to recipient, improving the conjugation efficiency to 1.3 × 10^−6^ (about 132 conjugants), which ensured the viability of subsequent experiments.

### Design of CRISPR/Cas9 vector for genome editing in *S. fradiae*

NeoI (NCBI ID: CP974_27380) is a repressor for structural genes (*neoE-D*) transcription, and deletion of *neoI* might enhance the transcription levels of target genes for neomycin synthesis in *S. fradiae*. In the beginning, the *neoI* deletion plasmid was constructed on the basis of pKC1139, named pKC-Δ*neoI* (Fig. S2A). Although the optimized conjugation conditions permitted pKC-Δ*neoI* to transfer in Sf01, we did not get any successfully edited *S. fradiae* strain but reverse mutation one after double crossovers.

**Fig 1:**
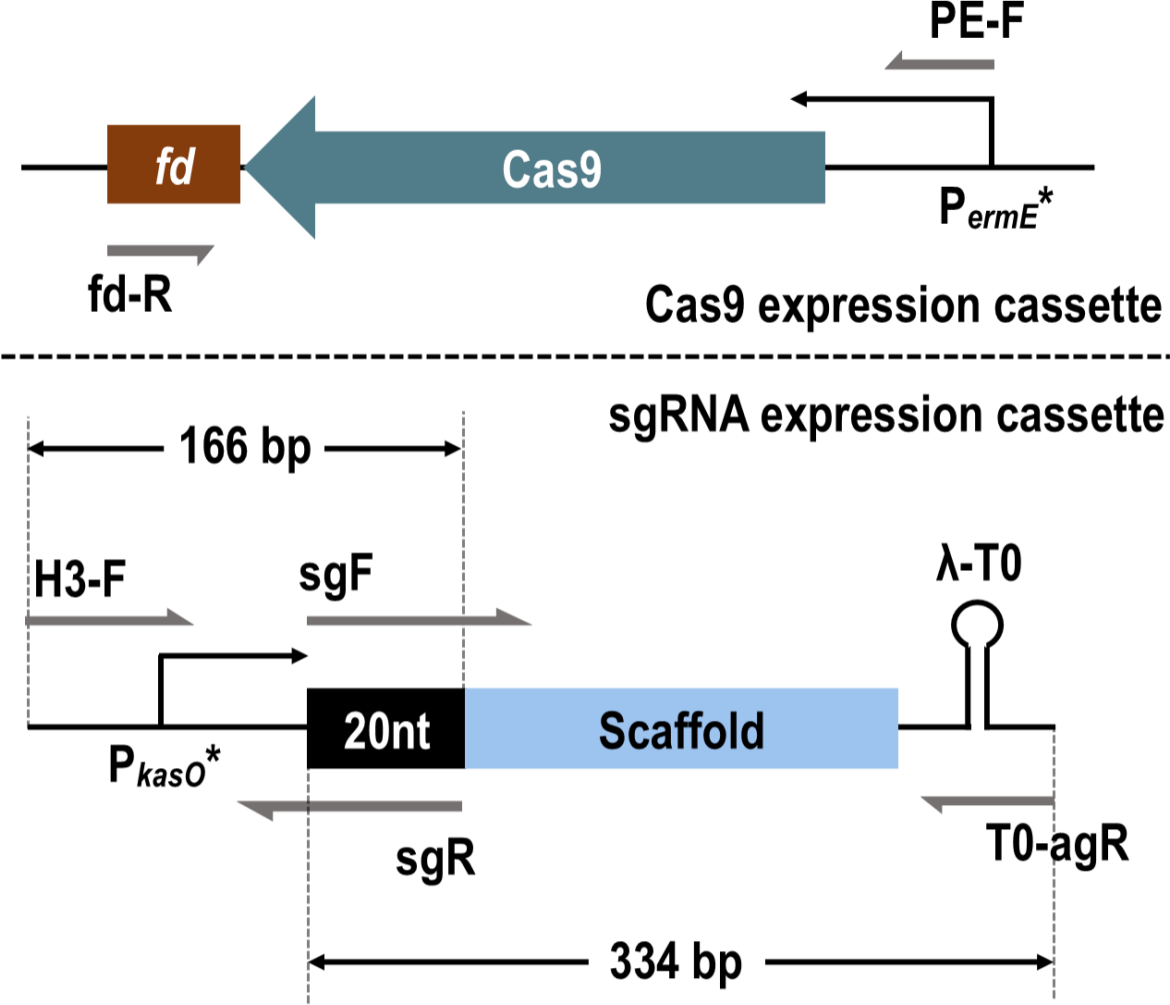
Expression cassette maps of Cas9 and sgRNA.

**Fig 2:**
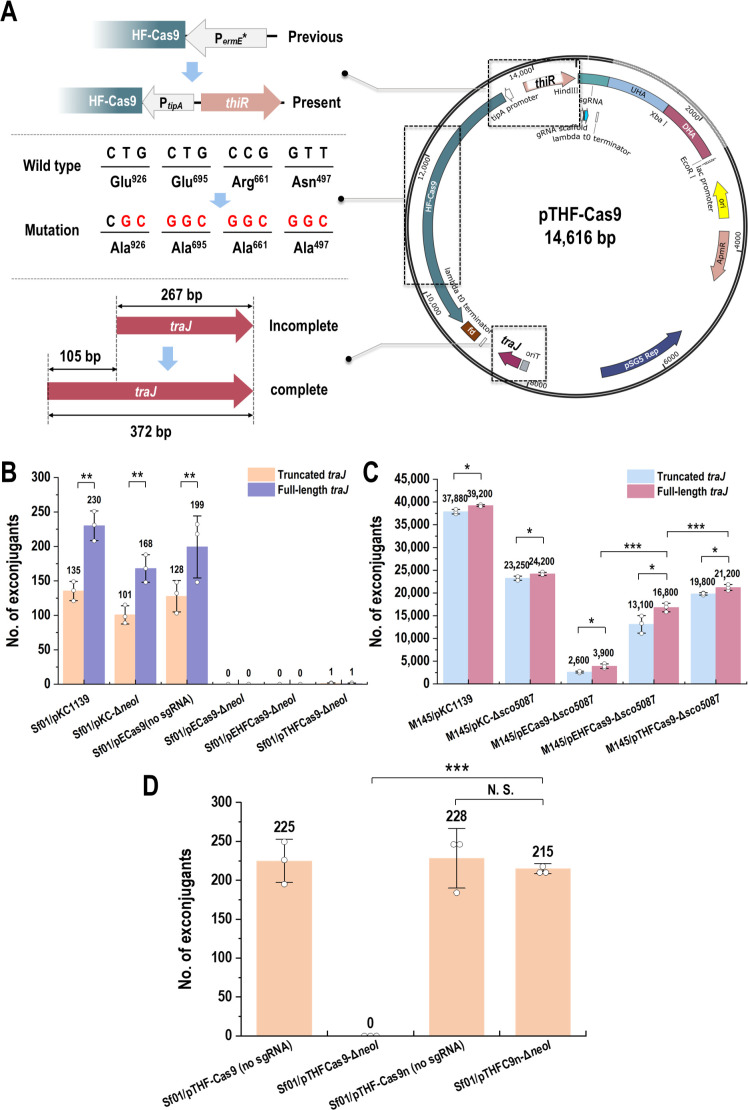
Construction and ability assessment of Cas9 mediated vectors. (**A**) pTHF-Cas9 plasmid map. (**B**) Conjugation efficiency of vectors in *S. fradiae* Sf01. (**C**) Conjugation efficiency of vectors in *S. coelicolor* M145. (D) The effect of D10A mutation introduction on conjugation efficiency (statistical analyses by *t*-test; * *P* < 0.05, ** *P* < 0.01, *** *P* < 0.001; N.S., no significant difference).

CRISPR/Cas9 represents the latest generation of gene editing tools, with a wide range of applications in *Streptomyces* (18, 21), and its efficacy in DNA transformation has been well documented. Here, the Cas9 editing vector was designed on the basis of pKC1139 by introducing the Cas9 and single-guided RNA (sgRNA) expressing cassette (Fig. 1). The Cas9 expression cassette included the Cas9 gene sequence with optimized codons according to the preference of *S. fradiae*, the constitutive promoter P_ermE_*, and FD terminator. sgRNA expression cassette included another constitutive promotor P*_kas_*_O_* (removed sequence of ribosome binding site) and λ-T0 terminator. The recombinant vector was named pECas9 (Fig. S2B). After designing the 20 nt for targeting *neoI* by CRISPy-web online, the sgRNA was linked with upstream and downstream homologous arms of *neoI* to construct the recombinant plasmid pECas9-Δ*neoI*. However, no conjugant was obtained after repeated conjugation. By comparing the number of conjugants, using pKC1139, pECas9 without sgRNA, and pECas9-Δ*neoI* as donor plasmids (Fig. 2B), the lack of sgRNA did not affect the conjugation efficiency, while the addition of sgRNA would severely impair the conjugation. This result was consistent with previous research (24, 25), which suggested that the “off-target effect” was caused by mismatches between the sgRNA-Cas9 complex and DNA strands, and should be blamed for the low conjugation efficiency.

### Optimization of Cas9 expression cassette to reduce the off-target rate

To achieve the conjugation, the following optimizations were made to the vector pECas9. Previous report has implied that the imperfection of TraJ (conjugation promoting factor) might influence conjugation efficiency (26). Consequently, the incomplete *traJ* in the vector pECas9 was modified by the full-length one. To reduce the frequency of the “off-target effect,” four high-fidelity mutations (N497A, R661A, Q695A, and Q926A) were introduced in the Cas9 DNA sequence. The four mutated amino acids, N497, R661, Q695, and Q926, were substituted by alanine at the location where they linked with the DNA strand through hydrogen bonds/salt bridges. It has been demonstrated that if a partial mismatch occurred between sgRNA and DNA, the cleavage activity of Cas9 would be inhibited. Cas9 carrying these four mutations had undetectable off-target events and its capacity for cutting DNA remained comparable to that of wild-type Cas9 (27). The recombinant plasmid was named pEHF-Cas9. Moreover, the constitutive promoter P_ermE_* was replaced by thiostrepton-inducible promoter P*_tip_*_A_ for Cas9 expression, correspondingly, thiostrepton resistance gene *thiR* was integrated in the vector, locating on the upstream of Cas9 expressing cassette and owning the opposite direction. The optimized vector was named pTHF-Cas9, and the optimization parts were summarized in Fig. 2A.

To verify the editing ability of pTHF-Cas9 in different *Streptomyces*, we designed pTHFCas9-Δ*neoI* for *S. fradiae* and pTHFCas9-Δ*sco5087* for *S. coelicolor* M145, respectively. A large number of transformants were shown in M145/pTHFCas9-Δ*sco5087*, while only one transformant was presented in Sf01/pTHFCas9-Δ*neoI*. The previous Cas9-mediated editing tools were also tested in *S. coelicolor* M145 for the deletion of *sco5087*. The introduction of four high-fidelity mutations did reduce the toxicity of Cas9 (Fig. 2C). However, this was also insufficient for Cas9-mediated gene editing tools to be applied in *S. fradiae*.

### Design of Cas9n-mediated editing vector and evaluating its editing efficiency

To reduce the negative impact of the “off-target effect,” D10A mutation was introduced in pEHF-Cas9 and pTHF-Cas9, and two reconstructed vectors were named pEHF-Cas9n and pTHF-Cas9n, respectively (Fig. S3 and S4).

**Fig 3:**
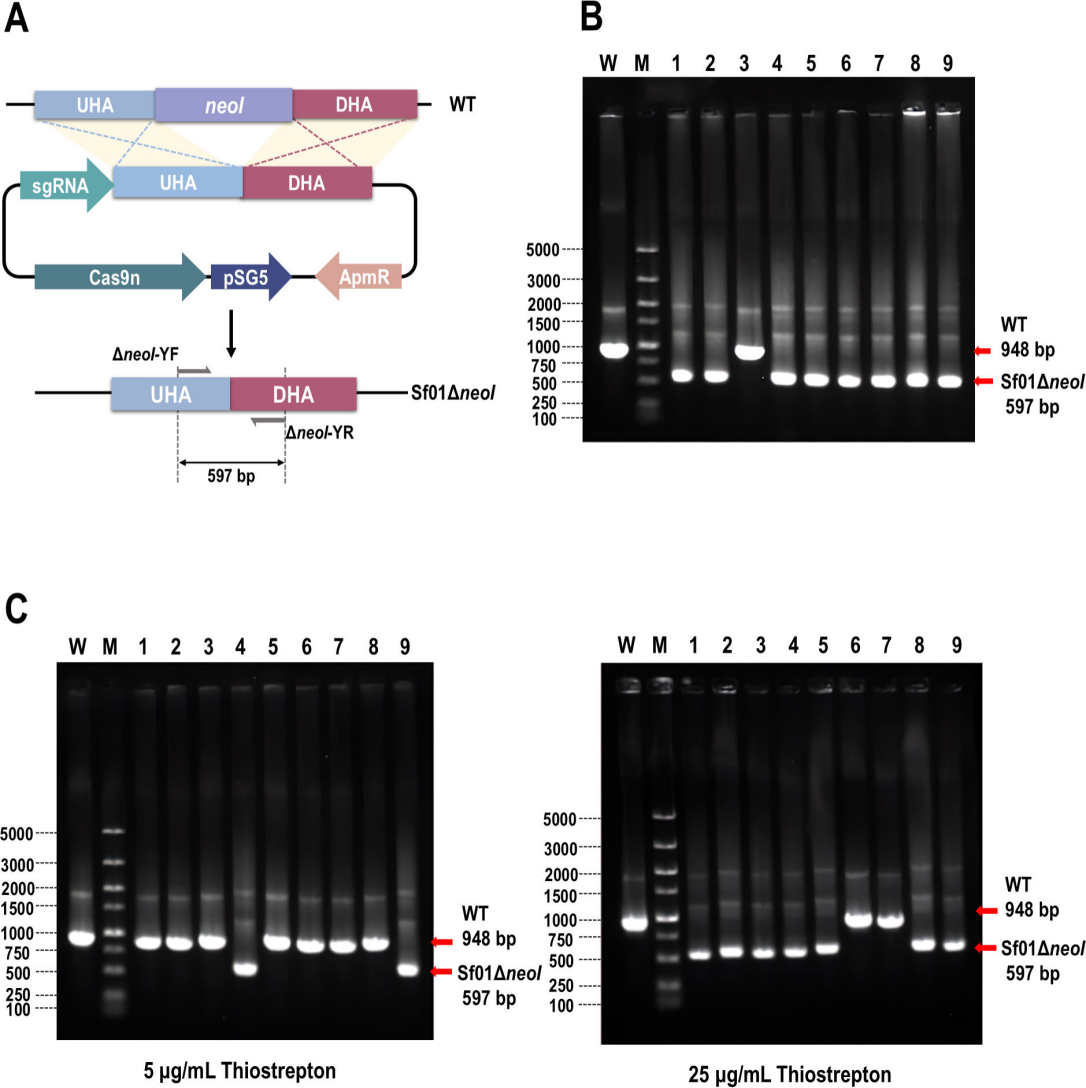
Evaluation of pEHF-Cas9n and pTHF-Cas9n for the construction of Sf01Δ*neoI*. (**A**) Editing processing of Sf01Δ*neoI* using pHF-Cas9n. (**B**) PCR validation of Sf01Δ*neoI* through pEHF-Cas9n. Line M, DL 5000 DNA marker (5,000, 3,000, 2,000, 1,500, 1,000, 750, 500, 250, and 100 bp); line W, PCR product of Sf01 using Δ*neoI*-YF/Δ*neoI*-YR, 948 bp; line 1–9, PCR product of Sf01Δ*neoI* using Δ*neoI*-YF/Δ*neoI*-YR, 597 bp for expected size. (**C**) Effect of thiostrepton concentration on the editing capability of pTHF-Cas9n.

On the evaluation of these two Cas9n-mediated editing vectors, gene *neoI* deletion vectors pEHFC9n-Δ*neoI* and pTHFC9n-Δ*neoI* were, respectively, constructed, and then applied to establish the recombined strains Sf01Δ*neoI* (Fig. 3A). The editing efficiencies of pEHFC9n-Δ*neoI* and pTHFC9n-Δ*neoI* were 88% and 77%, respectively (Fig. 3B and C).

Another comparison was made between the abilities of these two plasmids to knock out the large fragment, and the staurosporine biosynthetic gene cluster was designed as the target for gene deletion (Fig. S5). pTC9n-Δ*sta* and pEC9n-Δ*sta* were designed to knock out the *staN-staC* region (13.3 kb), while only pTC9n-Δ*sta* achieved the deletion strain, with 44% editing efficiency. Consequently, pTHF-Cas9n was accepted as the editing vector for gene editing in *S. fradiae*.

### Metabolic engineering of *S. fradiae* for high-level production of neomycin based on pTHF-Cas9n

#### Gene deletion

**Fig 4:**
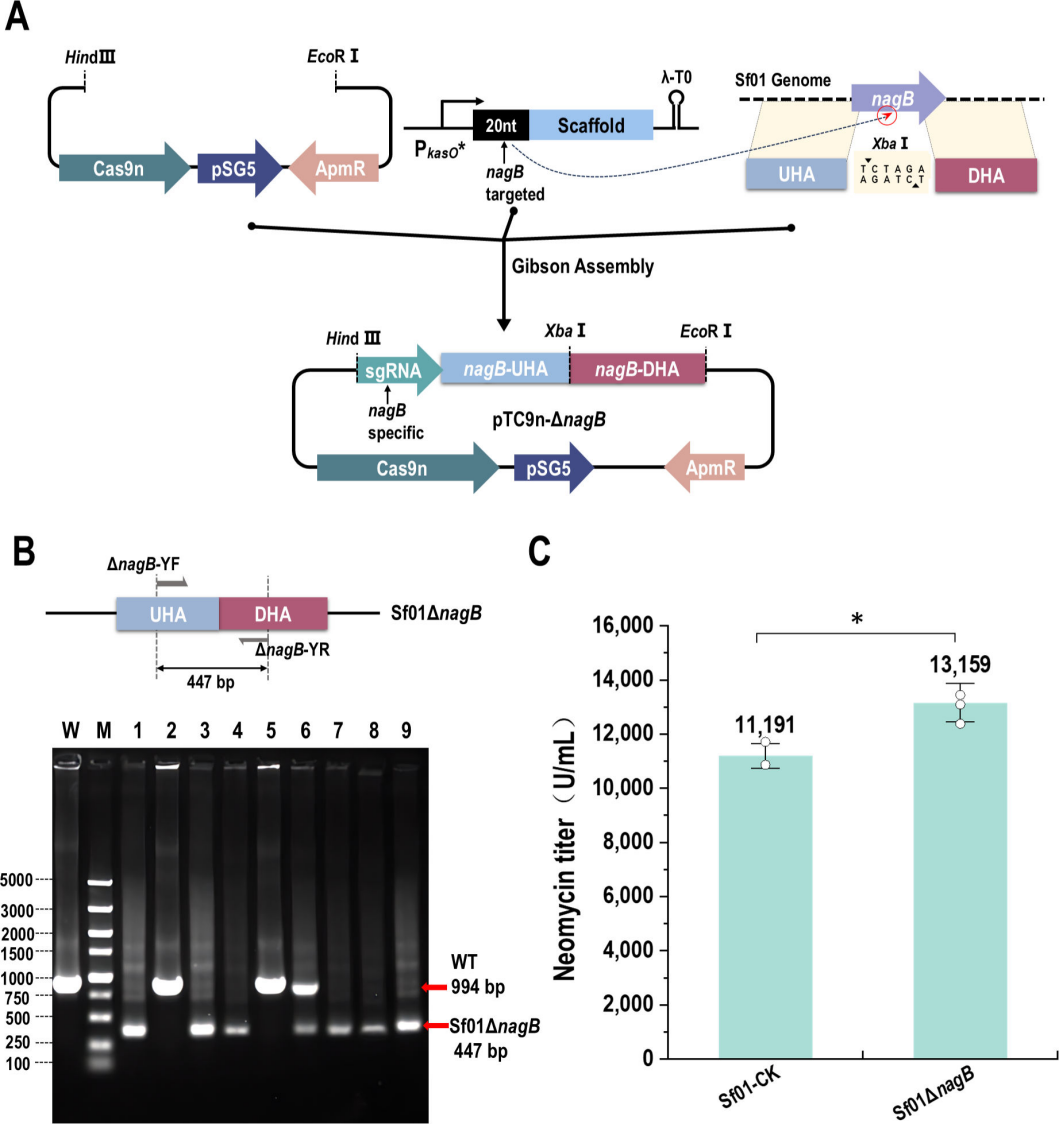
Deletion of gene *nagB* and its effect on neomycin production. (**A**) Assembly procession of pTC9n-Δ*nagB*. (**B**) PCR validation of Sf01Δ*nagB*. Line W, PCR product of Sf01 using Δ*nagB*-YF/Δ*nagB*-YR, 994 bp; line M, DL 5000 DNA marker (5,000, 3,000, 2,000, 1,500, 1,000, 750, 500, 250, and 100 bp); line 1–9, PCR product of Sf01Δ*nagB* using Δ*nagB*-YF/Δ*nagB*-YR, 447 bp for expected size. (**C**) Neomycin titer assessment (statistical analyses by *t*-test; * *P* < 0.05).

Glucosamine-6-phosphate deaminase NagB catalyzes the conversion of GlcN-6P to Fru-6P (28). The deletion of *nagB* might enhance UDP-GlcNAc concentration, thus improving neomycin production. pTC9n-Δ*nagB* was designed to construct gene *nagB* deletion strain, and the assembling process was shown in Fig. 4A. The result showed that *nagB* was knocked out successfully with the editing efficiency of 77.8% (Fig. 4B), and the recombinant strain was named as Sf01Δ*nagB*. The neomycin potency value of Sf01Δ*nagB* was 13,158.75 U/mL, which was 17.58% higher than that of Sf01 (11,191.29 U/mL) (Fig. 4C).

#### Gene-segment replacement

**Fig 5:**
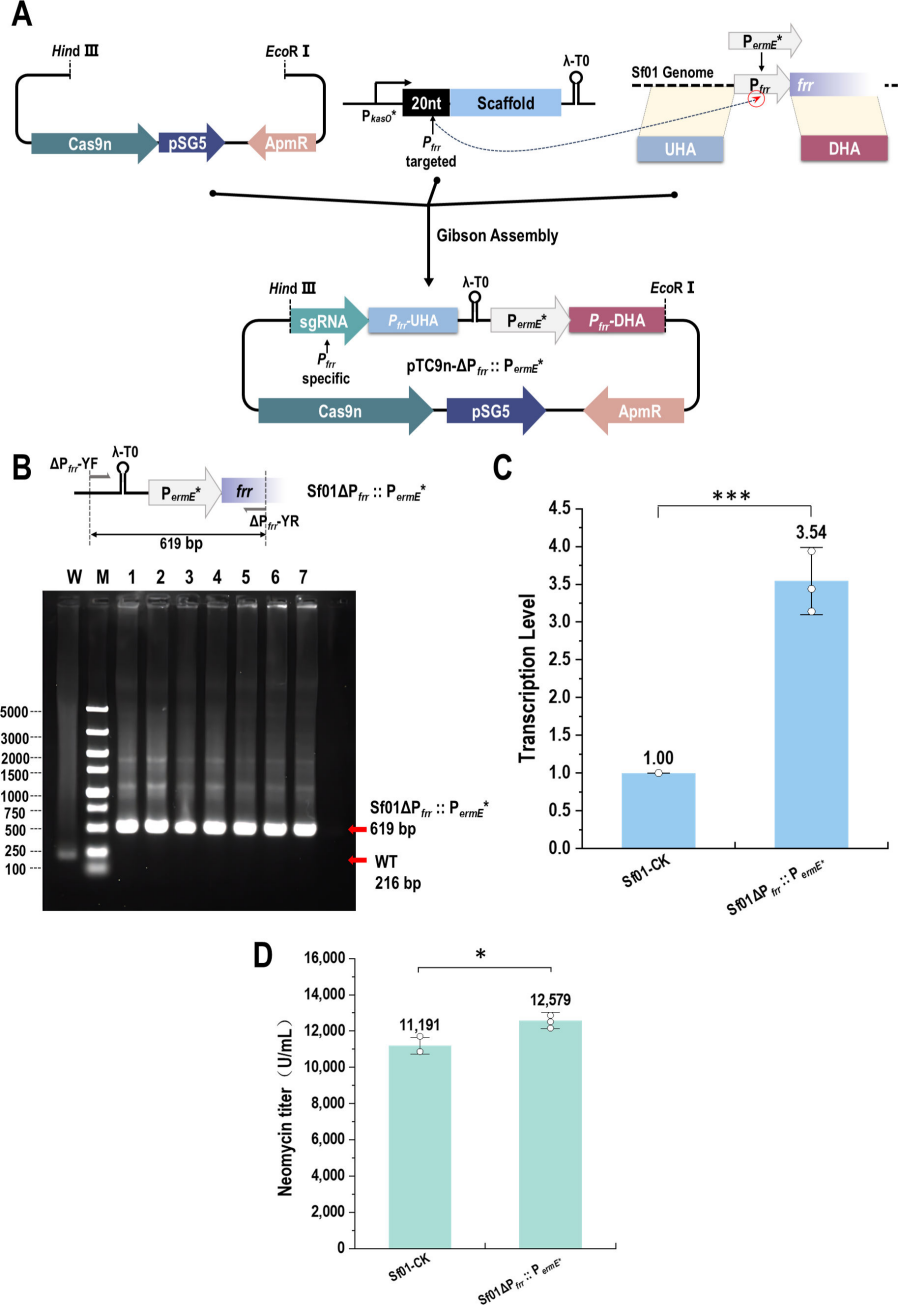
Replacement of *frr* promotor and its effect. (**A**) Assembly procession of pTC9n-ΔP*_frr_*::P_ermE_*. (**B**) PCR validation of Sf01ΔP*_frr_*::P_ermE_*. Line W, PCR product of Sf01 using P*_frr_*-YF/P*_frr_*-YR, 286 bp; line M, DL 5000 DNA marker (5,000, 3,000, 2,000, 1,500, 1,000, 750, 500, 250, and 100 bp); line 1–7, PCR product of Sf01ΔP*_frr_*::P_ermE_* using P*_frr_*-YF/P*_frr_*-YR, 619 bp for expected size. (**C**) Transcription levels of gene *frr*. (**D**) Neomycin titer assessment (statistical analyses by *t*-test; * *P* < 0.05, ** *P* < 0.01, *** *P* < 0.001).

Overexpression of *frr* (NCBI ID: CP974_22500, encoding for ribosome recycling factor) was reported to be beneficial for ribosome recycling (29). Therefore, we tended to improve the expression of FRR by replacing its natural promoter with a strong promotor P_ermE_*, and attained the vector pTC9n-ΔP*_frr_*::P_ermE_* (Fig. 5A). The recombinant strain Sf01ΔP*_frr_*::P_ermE_* was established with an efficiency of 100% (Fig. 5B), and transcription level of *frr* was 3.5-fold higher than the control strain (Fig. 5C). Additionally, neomycin potency value was 12,579.21 U/mL, which was 12.4% higher than that of Sf01 (Fig. 5D).

#### Integration of external gene cassette located on Δ*nagB*

**Fig 6:**
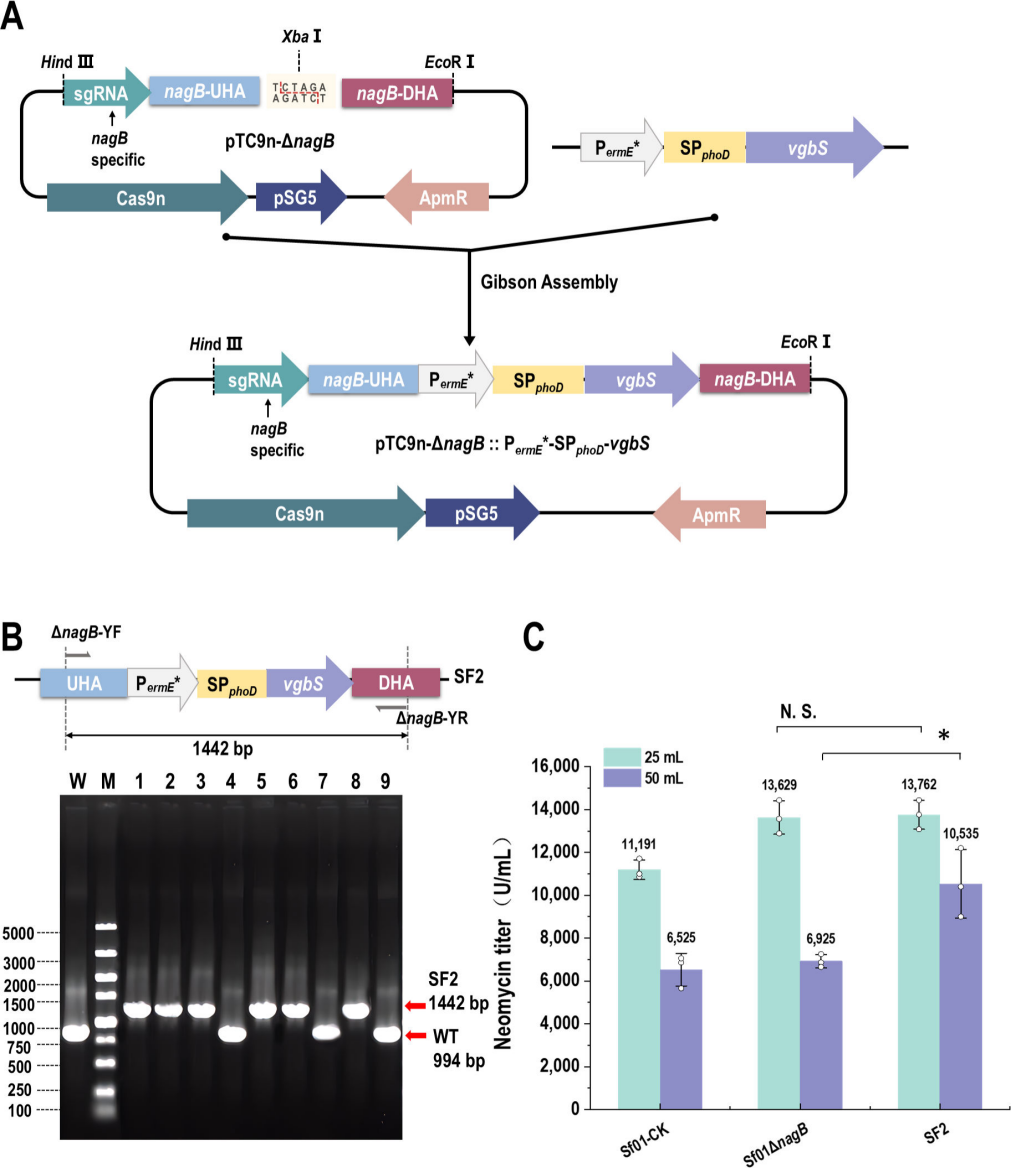
Integration of *vgbS* expression cassette and its effect on neomycin production. (**A**) Assembly procession of pTC9n-Δ*nagB*::P_ermE_*-SP*_phoD_-vgbS*. (**B**) PCR validation of SF2. Line W, PCR product of Sf01 using Δ*nagB*-YF/Δ*nagB*-YR, 994 bp; line M, DL 5000 DNA marker (5,000, 3,000, 2,000, 1,500, 1,000, 750, 500, 250, and 100 bp); line 1–7, PCR product of SF2 using Δ*nagB*-YF/Δ*nagB*-YR, 1,442 bp for expected size. (**C**) Neomycin titer assessment (statistical analyses by *t*-test; * *P* < 0.05; N.S., no significant difference).

The lack of oxygen in deep liquid fermentation limits the cell growth and secondary metabolism of *S. fradiae*, which brings a negative impact on neomycin synthesis. VHb is a hemoglobin from *Vitreoscilla*, and overexpression of its encoding gene *vgbS* could improve the absorption rate of oxygen in the anoxic situation (30). The *vgbS* expression cassette, including gene *vgbS*, promotor P_ermE_*, and signal peptide SP*_phoD_*, was inserted between upstream and downstream homologous arms of pTC9n-Δ*nagB*, resulting in the *vgbS* integration vector pTC9n-Δ*nagB*::P_ermE_*-SP*_phoD_-vgbS* (Fig. 6A). Then, gene *vgbS* expression cassette was introduced into Sf01 genome to replaced *nagB*, achieved the recombinant strain SF2 with the editing efficiency of 67.8% (Fig. 6B).

By comparing fermenting results between using the liquid media of 25 mL and 50 mL, discrepancies were presented in the group of 50 mL. While the group of 25 mL showed little difference between Sf01Δ*nagB* and SF2 (Fig. 6C). Therefore, the integration of the *vgbS* expression cassette was demonstrated to enhance neomycin production under the oxygen-limited condition, and have no adverse effects under the oxygen-rich condition.

## DISCUSSION

CRISPR/Cas9n editing tools have been extensively utilized for the genome modification of *Streptomyces*, however, there has been no documented evidence of the development or application of genome editing tools in *S. fradiae* using CRISPR/Cas9n. The majority of studies developing CRISPR editing tools in *Streptomyces* have employed model organisms as the experimental objects, with *S. coelicolor* being a particularly favored choice (17). The conjugation of *S. coelicolor* and *S. fradiae* with CRISPR/Cas9 editing vectors exhibits distinct states, demonstrating that *S. fradiae* exhibited high sensitivity to the toxicity of Cas9, indicating that the method of constructing recombinant strain of *S. coelicolor* using CRISPR/Cas9-mediated editing tool was incompatible in *S. fradiae. S. fradiae* (NCBI ID: ASM870442v1) has a GC content of 74.5% and is one of the *Streptomyces* with the highest GC content. In contrast, *S. coelicolor* M145 (NCBI ID: ASM893130v1) just has a GC content of 72%. It was postulated that GC content might be a determining factor in the compatibility of Cas9. A GC content of 74.5% led to an elevated off-target rate of Cas9 and a concomitant increase in the occurrence of double-strand breaks, thereby constraining the study and development of CRISPR/Cas9-based genome editing tool in *S. fradiae*. Nowadays, relative researchers have made great efforts to reduce the negative impact of “off-target effects,” including modifying the structure of Cas9 (27), regulating Cas9 expression (31), and improving the specificity of sgRNA (32). During the construction of the pTHF-Cas9 editing vector, four high-fidelity mutations were introduced in the initial vector pECas9. We simulated the interactions between the wild-type SpCas9 and the target DNA–sgRNA duplex, based on PDB accession 4OO8 and 4UN3 (27). By calculating the difference in required energy before and after the introduction of mutations, we found that the introduction of four mutations increased the free energy, thereby reducing the stability of the target DNA–sgRNA duplex (Table S1). It was speculated that the introduction of the four mutations ensured fidelity by reducing the binding stability between sgRNA and target DNA. By comparing the number of conjugants between M145/pECas9-Δ*sco5087* and M145/pEHFCas9-Δ*sco5087*, the introduction of four high-fidelity mutations did reduce the toxicity of Cas9 (Fig. 2C). Nevertheless, the diminished toxicity variant proved inadequate for the implementation of Cas9 mediated editing tool in *S. fradiae* until the D10A mutation was introduced. The D10A mutation replaced double-strand breaks with single-strand breaks, so pTHF-Cas9n showed a further decrease in toxicity (Fig. 2D). In addition, the editing efficiency of pEHF-Cas9n was higher than that of pTHF-Cas9n on the construction of Sf01Δ*neoI*, while only pTHF-Cas9n achieved large fragment knock out in Sf01. This was interpreted that the efficacy of genome editing via Cas9n-mediated tools was limited by the DNA repair capacity of the host strain. In Sf01, this limited capacity allowed continuous cutting of constitutive Cas9n when editing small fragments, simultaneously ensuring the proportion of positive colonies in transformants. However, this capacity was insufficient to repair the continuous cutting of Cas9n when editing large fragments, which resulted in failed editing. In contrast, genome cleavage was only possible in the presence of an inducer, guaranteeing a timely DNA repair. Although the proportion of positive colonies in the deletion of large fragments was less than half, pTHF-Cas9n shows better versatility than pEHF-Cas9n in genome editing of Sf01.

This enabled the development and application of a Cas9n-mediated genome editing tool in *S. fradiae* but also addressed a gap in genome editing in the *Streptomyces* family by providing a related editing tool.

CRISPR-mediated genome editing is a process that relies on the collaboration of three key components: the sgRNA, Cas9 endonuclease, and homologous arms. Editing vectors containing homologous arms between 0.5 kb and 2 kb always demonstrate proficiency in genome editing in Gram-positive bacteria (33, 34). The plasmid pTHF-Cas9n contained 1 kb homologous arms, which served as the template for DNA repair process, and its editing efficiency of P*_frr_* replacement (132 bp) was 100%, the *nagB* and *neoI* deletion (450 bp of *nagB* and 351 bp of *neoI*) were both 78%, the *vgbS* expression cassette integration (800 bp) was 67%, the *sta* gene cluster deletion (13.3 kb) was just 44%. The results above revealed an inverse relationship between the editing efficiency of pTHF-Cas9n and the size of edited DNA fragments, while the editing efficiency of large DNA fragments in *S. fradiae* can be improved by increasing the size of homologous arms. However, enlarging homologous arms increased the size of pTHF-Cas9n (exceeding 16 kb), potentially raising the bar for conjugation and genome editing, that cannot be ignored.

*Streptomyces* family has been responsible for approximately 70% of antibiotic production globally, representing a significant bioresource (5). To improve the biosynthetic capacity of the target antibiotic, the modification of *Streptomyces* included the reinforcement of precursor supply pathways (28, 35, 36), optimization of expression regulatory elements (37, 38), and improvement of energy utilization (39, 40). Neomycin is an aminoglycoside antibiotic produced by *S. fradiae*, and the above strategies were also applied to improve neomycin production in Sf01. The deletion of *nagB* in this study prevented the conversion of GlcN-6P to Fru-6P, thereby increasing the concentration of UDP-GlcNAc, which led to an improvement in neomycin yield, although it was previously reported that high concentrations of GlcN-6P caused weak growth (41). Furthermore, the effect of *nagB* deletion and *frr* overexpression on neomycin synthesis in *S. fradiae* was comparable, resulting in a 20% increase in neomycin production relative to the original strain. Furthermore, the integration of the *vgbS* cassette resulted in an enhanced synthesis level of neomycin. These strains served as reliable neomycin-producing strains for subsequent gene stacking and modification of engineering strains, laying the foundation for subsequent increases in industrial production of neomycin.

Taken together, the development of pTHF-Cas9n for genome editing in *S. fradiae* offers a successful case of applying CRISPR/Cas9n in the *Streptomyces* family. This study also demonstrates the feasibility of CRISPR/Cas9n-mediated genome editing tools in organisms with high GC content. Moreover, our study has also facilitated the construction of robust and stable engineering strains for the enhancement of antibiotic production.

## MATERIALS AND METHODS

### Bacterial strains and culture conditions

The strains and plasmids used in this study are listed in Table 1. S*. fradiae* Sf01 served as the initial strain for the construction of recombinant strains. *Escherichia coli* XL10-Gold was used to construct recombinant plasmids, and ET12567/pUZ8002 was used as a donor for conjugation.

**TABLE 1:** Strains and plasmids used in this study

Strains or plasmids	Description	Source
Strains		
*S. fradiae* Sf01	Wild type	Stored in lab
*S. fradiae* Sf01Δ*neoI*	Sf01Δ*neoI*	This study
*S. fradiae* Sf01Δ*nagB*	Sf01Δ*nagB*	This study
*S. fradiae* Sf01ΔP*_frr_*::P_ermE_*	Sf01ΔP*_frr_*::P_ermE_*	This study
*S. fradiae* SF2	Sf01Δ*nagB*::P_ermE_*-SP*_phoD_-vgbS*	This study
*S. coelicolor* M145	Wild type	(42)
*E. coli* XL10-Gold	Plasmid construction, weak Tet^r^	Stratagene, US
*E. coli* ET12567/pUZ8002	Conjugation intermediate, demethylated strain containing plasmid pUZ8002, Chl^r^, Kan^r^	(43)
Plasmids		
pSET152	Integrated vector with *attP* from φC31 located on *attB*; Apm^r^	(44)
pKC1139 (truncated *traJ*)	Streptomyces genome editing vector, Apm^r^	(45)
pCas9	Genome editing vector, Kan^r^	(22)
pKC1139 (full-length *traJ*)	Streptomyces genome editing vector, Apm^r^	This study
pKC-Δ*neoI* (truncated *traJ*)	pKC1139 + *Hin*dIII + 1 kb *neoI-*UHA + *Xba*I + 1 kb *neoI-*DHA + *Eco*RI, Apm^r^	This study
pKC-Δ*neoI* (full-length *traJ*)	pKC1139 + *Hin*dIII + 1 kb *neoI-*UHA + *Xba*I + 1 kb *neoI-*DHA + *Eco*RI, Apm^r^	This study
pECas9 (truncated *traJ*)	pKC1139 + P_ermE_* + Cas9 + T*_fd_ + Hin*dIII + P_kasO_* + sgRNA (no target sequence) + *Eco*RI, Apm^r^	This study
pECas9 (full-length *traJ*)	pKC1139 + P_ermE_* + Cas9 + T*_fd_ + Hin*dIII + P_kasO_* + sgRNA (no target sequence) + *Eco*RI, Apm^r^	This study
pECas9-Δ*neoI* (truncated *traJ*)	pECas9 + *Hin*dIII + P_kasO_* + sgRNA (target *neoI*) + 1 kb *neoI-*UHA + *Xba*I + 1 kb *neoI-*DHA + *Eco*RI, Apm^r^	This study
pECas9-Δ*neoI* (full-length *traJ*)	pECas9 + *Hin*dIII + P_kasO_* + sgRNA (target *neoI*) + 1 kb *neoI-*UHA + *Xba*I + 1 kb *neoI-*DHA + *Eco*RI, Apm^r^	This study
pTHF-Cas9 (truncated *traJ*)	pKC1139 + P*_tipA_* + HF-Cas9 + T*_fd_ + Hin*dIII + P_kasO_* + sgRNA (no target sequence) + *Eco*RI, Thi^r^, Apm^r^	This study
pTHF-Cas9 (full-length *traJ*)	pKC1139 + P*_tipA_* + HF-Cas9 + T*_fd_ + Hin*dIII + P_kasO_* + sgRNA (no target sequence) + *Eco*RI, Thi^r^, Apm^r^	This study
pTHFCas9-Δ*neoI* (truncated *traJ*)	pTHF-Cas9 + *Hin*dIII + P_kasO_* + sgRNA (target *neoI*) + 1 kb *neoI-*UHA + *Xba*I + 1 kb *neoI-*DHA + *Eco*RI, Thi^r^, Apm^r^	This study
pTHFCas9-Δ*neoI* (full-length *traJ*)	pTHF-Cas9 + *Hin*dIII + P_kasO_* + sgRNA (target *neoI*) + 1 kb *neoI-*UHA + *Xba*I + 1 kb *neoI-*DHA + *Eco*RI, Thi^r^, Apm^r^	This study
pKC-Δsco*5087* (truncated *traJ*)	pKC1139 + *Hin*dIII + 1 kb sco*5087-*UHA + *Xba*I + 1 kb sco*5087-*DHA + *Eco*RI, Apm^r^	This study
pKC-Δsco*5087* (full-length *traJ*)	pKC1139 + *Hin*dIII + 1 kb sco*5087-*UHA + *Xba*I + 1 kb sco*5087-*DHA + *Eco*RI, Apm^r^	This study
pECas9-Δsco*5087* (truncated *traJ*)	pECas9 + *Hin*dIII + P_kasO_* + sgRNA (target sco*5087*) + 1 kb sco*5087-*UHA + *Xba*I + 1 kb sco*5087-*DHA + *Eco*RI, Apm^r^	This study
pECas9-Δsco*5087* (full-length *traJ*)	pECas9 + *Hin*dIII + P_kasO_* + sgRNA (target sco*5087*) + 1 kb sco*5087-*UHA + *Xba*I + 1 kb sco*5087-*DHA + *Eco*RI, Apm^r^	This study
pEHFCas9-Δsco*5087* (truncated *traJ*)	pEHF-Cas9 + *Hin*dIII + P_kasO_* + sgRNA (target sco*5087*) + 1 kb sco*5087-*UHA + *Xba*I + 1 kb sco*5087-*DHA + *Eco*RI, Apm^r^	This study
pEHFCas9-Δsco*5087* (full-length *traJ*)	pEHF-Cas9 + *Hin*dIII + P_kasO_* + sgRNA (target sco*5087*) + 1 kb sco*5087-*UHA + *Xba*I + 1 kb sco*5087-*DHA + *Eco*RI, Apm^r^	This study
pTHFCas9-Δsco*5087* (truncated *traJ*)	pTHF-Cas9 + *Hin*dIII + P_kasO_* + sgRNA (target sco*5087*) + 1 kb sco*5087-*UHA + *Xba*I + 1 kb sco*5087-*DHA + *Eco*RI, Thi^r^, Apm^r^	This study
pTHFCas9-Δsco*5087* (full-length *traJ*)	pTHF-Cas9 + *Hin*dIII + P_kasO_* + sgRNA (target sco*5087*) + 1 kb sco*5087-*UHA + *Xba*I + 1 kb sco*5087-*DHA + *Eco*RI, Thi^r^, Apm^r^	This study
pEHF-Cas9n	pKC1139 + P_ermE_* + HF-Cas9*n* + T*_fd_ + Hin*dIII + P_kasO_* + sgRNA (no target sequence) + *Eco*RI, Apm^r^	This study
pTHF-Cas9n	pKC1139 + P*_tipA_* + HF-Cas9*n* + T*_fd_ + Hin*dIII + P_kasO_* + sgRNA (no target sequence) + *Eco*RI, Thi^r^, Apm^r^	This study
pEHFC9n-Δ*neoI*	pEHF-Cas9*n* + *Hin*dIII + P_kasO_* + sgRNA (target *neoI*) + 1 kb *neoI-*UHA + *Xba*I + 1 kb *neoI-*DHA + *Eco*RI, Apm^r^	This study
pTHFC9n-Δ*neoI*	pTHF-Cas9*n* + *Hin*dIII + P_kasO_* + sgRNA (target *neoI*) + 1 kb *neoI-*UHA + *Xba*I + 1 kb *neoI-*DHA + *Eco*RI, Thi^r^, Apm^r^	This study
pEC9n-Δ*sta*	pEHF-Cas9*n* + *Hin*dIII + P_kasO_* + sgRNA (target *sta*) + 1 kb *sta-*UHA + *Xba*I + 1 kb *sta-*DHA + *Eco*RI, Thi^r^, Apm^r^	This study
pTC9n-Δ*sta*	pTHF-Cas9*n* + *Hin*dIII + P_kasO_* + sgRNA (target *sta*) + 1 kb *sta-*UHA + *Xba*I + 1 kb *sta-*DHA + *Eco*RI, Thi^r^, Apm^r^	This study
pTC9n-Δ*nagB*	pTHF-Cas9*n* + *Hin*dIII + P_kasO_* + sgRNA (target *nagB*) + 1 kb *nagB-*UHA + *Xba*I + 1 kb *nagB-*DHA + *Eco*RI, Thi^r^, Apm^r^	This study
pTC9n-ΔP*_frr_*::P_ermE_*	pTHF-Cas9*n* + *Hin*dIII + P_kasO_* + sgRNA (target P*_frr_*) + 1 kb P*_frr_-*UHA + (λ-T0 + P_ermE_*) + 1 kb P*_frr_-*DHA + *Eco*RI, Thi^r^, Apm^r^	This study
pTC9n-Δ*nagB*::P_ermE_*-SP*_phoD_-vgbS*	pTC9nΔ*nagB +* Δ*Xba*I::(P_ermE_* + SP*_phoD_ +* vgbS) Thi^r^, Apm^r^	This study

LB soil medium (1% tryptone, 0.5% yeast extract, 1% NaCl, and 2% agar, pH 7.2) was used for the cultivation of *E. coli*. SFMM soil medium (2% soybean cake powder hydrolysate, 2% mannitol, 50 mM MgCl_2_, 2% agar, pH 7.5) was used for the cultivation of *S. fradiae* and *S. coelicolor*. Antibiotics (50 mg/L apramycin, 25 mg/L kanamycin, 25 mg/L chloramphenicol, 25 mg/L naphthoquinone acid, or 25 mg/L thiostreptone) were added into the media as required. *E. coli* was cultivated at 37°C, while *S. fradiae* and *S. coelicolor* were cultivated at 30°C. For neomycin production, the seed was cultivated in 25 mL TSBY medium (3% trypticase soy broth, 0.5% yeast extract, 1% NaCl, pH 7.2) for 18 h, and then transferred into neomycin fermentation medium (8% corn starch, 3% peanut cake powder, 2% glucose, 1.2% peptone, 0.6% yeast powder, 0.6% (NH_4_)_2_SO_4_, 0.45% NaCl, pH 7.2–7.5) for 7 days. All of the fermentation experiments were performed in triplicate.

The formulation of other culture media for testing the effectiveness of *S. fradiae* cultivation is listed as follows. IWL4: 1% soluble starch, 0.1% K_2_HPO_4_, 0.1% NaCl, 0.2% (NH_4_)_2_SO_4_, 0.2% CaCO_3_, 0.4% tryptone, 0.1% yeast extract, 0.1% MgSO_4_·7 H_2_O, 0.8% MgCl_2_, 2% agar, pH 7.2; 2CMY: 1% soluble starch, 0.2% tryptone, 0.1% NaCl, 0.2% K_2_HPO_4_, 0.2% CaCO_3_, 2% agar, pH 7.2; GS-1: 2% soluble starch, 0.1% KNO_3_, 0.05% K_2_HPO_4_, 0.05% MgSO_4_·7 H_2_O, 0.001% FeSO_4_·7H_2_O, 0.05% NaCl, 2% agar, pH 7.4; ISP2: 1% malt extract, 0.4% yeast extract, 0.4% glucose, 2% agar, pH 7.2.

### Construction of editing plasmids

All primers used in this study are listed in Table 2. To construct pKC-Δ*neoI*, pKC1139 was double digestion with *Hin*d III and *Eco*R I, obtaining a linearized vector. Homologous arms were obtained by PCR amplification with pKCΔ*neoI*-UF/pKCΔ*neoI*-UF and pKCΔ*neoI*-DF/pKCΔ*neoI*-DF using the Sf01 genome as the template. 2× MultiF Seamless Assembly Mix (ABclonal Technology) was used to assemble all of the linearized vector and DNA segments in this study.

**TABLE 2:** Primers used in this study

Primers	Sequence (5’–3’)
pKCΔ*neoI*-UF	TAAAACGACGGCCAGTGCCAAGCTTGCCAGCGCACCGACCTTGA
pKCΔ*neoI*-UR	GTTCTGCACCCTGAGCCGCTGGTCGAACCTCC
pKCΔ*neoI*-DF	GACCAGCGGCTCAGGGTGCAGAACCTCGCATC
pKCΔ*neoI*-DR	ACAGCTATGACATGATTACGAATTCCCTTCGACGGCCATGAGGTA
Δ*neoI*-YF	CCGTGCCAGACGCCCTTGT
Δ*neoI*-YR	CGGCTTCGTTATGGACAACCC
H3-F	TAAAACGACGGCCAGTGCCAAGCTT
T0-agR	TCCAGTAATGACCTCAGAACTCCATC
Δ*neoI*-sgR	GTCGTCTGCGCTCGTCCGGAGGCCACGACTTTACAACACC
Δ*neoI*-sgF	TCCGGACGAGCGCAGACGACGTTTTAGAGCTAGAAATAGCAAG
PE-F1	CGTCGTGACTGGGAAAACCCTGCGGTCGATCTTGACGGCT
fd-R	GTTATGTTGATCGGCACTTTGAGCCTCAGCGATCGAATATA
*traJ*-GF	CGCTATAATGACCCCGAAGCAG
*traJ*-GR	GTTCGGTGATGCCACGATCC
*traJ*-F	CTGCTTCGGGGTCATTATAGCG
*traJ*-R	GGATCGTGGCATCACCGAAC
P*tipA*-F	CGTCGTGACTGGGAAAACCCTTTATCGGTTGGCCGCGAGATT
P*tipA*-R	ATGGAGTACTTCTTGTCCATATGTCCGCTCCCTTCTCTGA
Cas9-F	ATGGACAAGAAGTACTCCATCG
N497A-R	CTTGTCGAAGGCAGTCATCCGCTCAATGAAGG
N497A-F	CGGATGACTGCCTTCGACAAGAACCTGCCTAAC
R661A-R	TGCGGGAGAGGGCGCCCCACCCGGTGTACCGC
R661A-F	GGTGGGGCGCCCTCTCCCGCAAGCTGATAAACG
Q695A-R	TGGATGAGGGCCATGAAGTTACGGTTCGCGAA
Q695A-F	TAACTTCATGGCCCTCATCCACGACGACTCCCT
Q926A-R	GCTTGGTGATCGCTCGCGTTTCTACCAGCTGGC
Q926A-F	GAAACGCGAGCGATCACCAAGCACGTCGCGC
D10A-R	CCGACCGAGTTGGTGCCGATCGCGAGGCCGATGGAGTAC
D10A-F	ACTCCATCGGCCTCGCGATCGGCACCAACTCGGTCGGGTG
Δ*sta*-sgR	AATCCCCGCGAGTGCGGAGCGGCCACGACTTTACAACACC
Δ*sta*-sgF	GCTCCGCACTCGCGGGGATTGTTTTAGAGCTAGAAATAGCAAG
Δ*sta*-UF	GAGAGAGAGAGAGGAGAGAGAGCCCGCACCTGCCGATGCTC
Δ*sta*-UR	CCTCCGTCTAGAGACACGCCACCGCCCTGTCGTC
Δ*sta*-DF	GGCGTGTCTCTAGACGGAGGAGGTCGCCAAGCTCT
Δ*sta*-DR	ACAGCTATGACATGATTACGAATTCACAGCTTCACCGAGGGCATG
Δ*sta*-YF	CCGCACGCCACCTGTTCATC
Δ*sta*-YR	GAGGAGCTGTTCGCCCATGT
Δ*nagB*-sgR	GAGGGGGAAAGGGAGTCACAGGCCACGACTTTACAACACC
Δ*nagB*-sgF	TGTGACTCCCTTTCCCCCTCGTTTTAGAGCTAGAAATAGCAAG
Δ*nagB*-UF	GTTCTGAGGTCATTACTGGAGAGCTGCCACTCCGAGACC
Δ*nagB*-UR	GCGATCCTCTAGAGTCTCCGCCCCAGACCCCCCTTCG
Δ*nagB*-DF	CGGAGACTCTAGAGGATCGCACCCGCATCAAGACCCT
Δ*nagB*-DR	ACAGCTATGACATGATTACGAATTCCACCCCCGTGGCCAACATC
Δ*nagB*-YF	CGCGCAGCCGCCTGACGGA
Δ*nagB*-YR	AAGTACCCGGCGAGCTTCA
P*_frr_*-sgR	GGACAAACCCCAAGACACGCGGCCACGACTTTACAACACC
P*_frr_*-sgF	GCGTGTCTTGGGGTTTGTCCGTTTTAGAGCTAGAAATAGCAAG
P*_frr_*-UF	GTTCTGAGGTCATTACTGGACATCAAGGTCGGCATCTGAGCC
P*_frr_*-UR	GCCTTATTGTTGCGGATCCTTCTTGTCCTGCACGGTGTC
PE-F	AGGATCCGCAACAATAAGGC
PE-R	TATATATTCCTCCTTTCTAATATACCTG
P*_frr_*-DF	TTAGAAAGGAGGAATATATAGTGGTGATCGAAGAGACCCTCC
P*_frr_*-DR	ACAGCTATGACATGATTACGAATTCGGCCTTCTGGACGAAGAGC
P*_frr_*-YF	TCACCCTGTGCCGCGACAAC
P*_frr_*-YR	TCGGCTTCGAGGAGGGTCTCT
PE-BF	GGGGGGTCTGGGGCGGAGACAGGATCCGCAACAATAAGGC
*vgbS*-F	GTATATTAGAAAGGAGGAATATATAATGCTGGACCAGCAGACCAT
*vgbS*-R	TCTTGATGCGGGTGCGATCCTCACTCGACGGCCTGGGCGT
SP*_PhoD_*-F	TTAGAAAGGAGGAATATATAGTGACCAGTCGAAACCTCGTTCC
SP*_PhoD_*-R	GCCCGGCAGCTCCGGGCCCGGCAGGGGGGCGGAGGTGGTCGC
RT-*sigF*-F	TACCAGTACGTCCGGAACAC
RT-*sigF*-R	GCTTGATCTCGCCGACCA
RT-*frr*-F	TGTTCAACAAGATCGTGGCC
RT-*frr*-R	CCTTGGCGACCTTGATGTAC

Cas9 and Cas9n-mediated editing vectors were designed on the basis of pKC1139. Using the construction of pECas9-Δ*neoI* as an example. Linearized vector was obtained by pKCΔ*neoI* digestion with *Hin*d III. Cas9 expression cassette was obtained by amplification with PE-F1/fd-R using pCas9 as the template, and its expression direction was from the *Hin*d III digestion site to *traJ*. As for the sgRNA expression cassette, a specific 20 nt of sgRNA was designed by CRISPy-web (https://crispy.secondarymetabolites.org/#/input). Two fragments of sgRNA (*neoI*) were obtained by amplification with H3-F/Δ*neoI*-sgR, Δ*neoI*-sgF/T0-agR, and pCas9, then using SOE-PCR with H3-F/T0-agR to link a whole sgRNA expression cassette, and its expression direction was from *Hin*d III digestion site to homologous arms of pKC-Δ*neoI.*

Elements optimization was done on the basis of pECas9. Completed *traJ* was obtained by amplification with *traJ*-F/*traJ*-R using pSET152 as the template, while the linearized vector used pECas9 as the template and *traJ*-GF/*traJ*-GR as primers for amplification. Next, four mutants were introduced using Cas9-F/N497A-R, N497A-F/R661A-R, R661A-F/Q695A-R, Q695A-F/Q926A-R, Q926A-F/fd-R as primers, using pECas9 as template, and SOE-PCR was used to link five fragments with Cas9-F/fd-R, reconnecting Cas9 with HFCas9. The artificial synthesized fragment including promotor P*_tipA_* and *thiR* gene was used P*tipA*-F/P*tipA*-R for amplification, followed by a replacement with P_ermE_*.

The introduction process of the D10A mutation was similar to the introduction flow of the four mutants above. Here, the construction of the Cas9n-mediated editing plasmid showed the construction of pTC9n-Δ*nagB* as an example. sgRNA (*nagB*) expression cassette obtained from SOE-PCR of two fragments which used H3-F/Δ*nagB*-sgR, Δ*nagB*-sgF/T0-agR and pECas9 for PCR amplification and H3-F/T0-agR was used as primers for SOE-PCR. Homologous arms were obtained by PCR amplification with Δ*nagB*-UF/Δ*nagB*-UF and Δ*nagB*-DF/Δ*nagB*-DF using the Sf01 genome as the template. *Xba* I digestion site was induced in pTC9n-Δ*nagB* and located between the upward homologous arm and the downward homologous arms of *nagB*. Furthermore, a gene segment integration to replace *nagB* would use linearized pTC9n-Δ*nagB* digested by *Xba* I to construct an editing plasmid.

### Construction of *S. fradiae* strains

Sf01 spores as receptors were washed twice with TES buffer (0.05 M TES, pH 8.0), and then heated at 50°C for 10 min. Treated spores were suspended at TES buffer and preincubated at 30°C for 3 h. ET12567 as a donor was inoculated in LB medium at 37°C for 20 h. Then, ET12567 was collected and washed twice with LB medium. Next, a mixed donor with receptor (20:1), was culturing in SFMM medium at 30°C for 5–7 days. Nalidixic acid and apramycin were added within 18–20 h after conjugation.

### Plasmid elimination

Colonies were cultivated at 45°C, followed by an antibiotic sensitivity check. Apramycin-sensitive colonies were verified by colony PCR and Sanger sequencing.

### Editing efficiency calculation

The editing efficiency (%) = (the number of successfully edited colonies/total number of colonies containing the edited vector) × 100%. Specifically, after conjugation, all of the conjugants were grown in a medium containing apramycin, which ensured that the editing vector was transferred to the target. Then, these colonies with positive resistance to apramycin were induced to express Cas9n in another medium carrying apramycin and thiostreptone. Finally, colonies will be validated by comparing the consistency of PCR products with the expected size, followed by calculating the number of edited colonies.

### Transcription level analysis

Mycelium in the logarithmic phase was lysed by lysozyme, followed by RNA extraction via RNA isolation kit (FORE GENE), and extracting steps referred to in the user manual. Total RNA was treated with UnionScript First-strand cDNA mix kit (ABclonal Technology), and quantitative real-time PCR reactions were performed on the Applied Biosystems Step-One Plus system with Maxima SYBR Green/ROX qPCR Master Mix (ABclonal Technology), and gene ***sigF*** was used as the internal control to normalize samples.

### Neomycin quantiﬁcation

Neomycin titer was determined by Agilent HPLC-ELSD, using the Gzflm Titan F5 chromatographic column (2.1 mm × 150 mm, 3 µm), with 0.5%–0.7% trifluoroacetic acid solution as mobile phase, mobile phase flow rate of 0.15 mL/min, column temperature of 30°C, injection volume of 5 µL. The drift tube temperature was 75°C, and the carrier gas flow rate was 1.8 L/min.

## Data Availability

The data sets generated during and/or analyzed during the current study are available from the corresponding author upon reasonable request.

## SUPPLEMENTAL MATERIAL

Fig. S1 aem.01953-24-s0001.tiff Optimization of conjugant parameters for S. fradiae Sf01.

Fig. S2 aem.01953-24-s0002.tiff Construction of pKC-ΔneoI, and pECas9 plasmid map.

Fig. S3 aem.01953-24-s0003.tiff pEHF-Cas9n plasmid map.

Fig. S4 aem.01953-24-s0004.tiff pTHF-Cas9n plasmid map.

Fig. S5 aem.01953-24-s0005.tiff Construction of Sf01Δsta.

Supplemental material aem.01953-24-s0006.doc Table S1; legends for Fig. S1 to S5.

ASM does not own the copyrights to Supplemental Material that may be linked to, or accessed through, an article. The authors have granted ASM a non-exclusive, world-wide license to publish the Supplemental Material files. Please contact the corresponding author directly for reuse.
