# Supplementary figures and images for: Optimizing genome editing efficiency in Streptomyces fradiae via a CRISPR/Cas9n-mediated editing system
Source: Appl Environ Microbiol. 2025 Jan 22;91(2):e01953-24. doi: 10.1128/aem.01953-24 (PMC11837490; doi:10.1128/aem.01953-24)

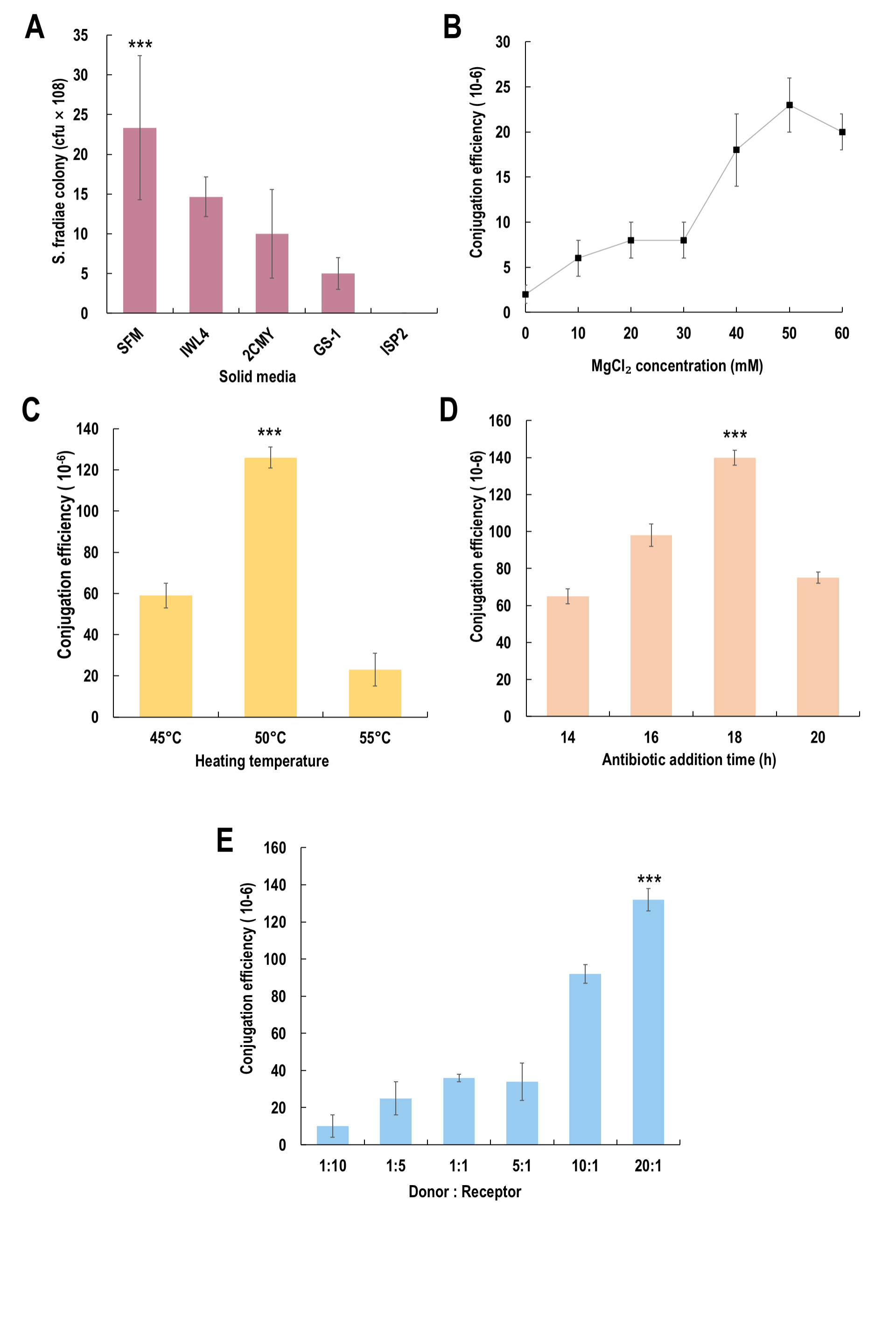

Supplement: Fig. S1 — Optimization of conjugant parameters for S. fradiae Sf01. [file aem.01953-24-s0001.tiff]

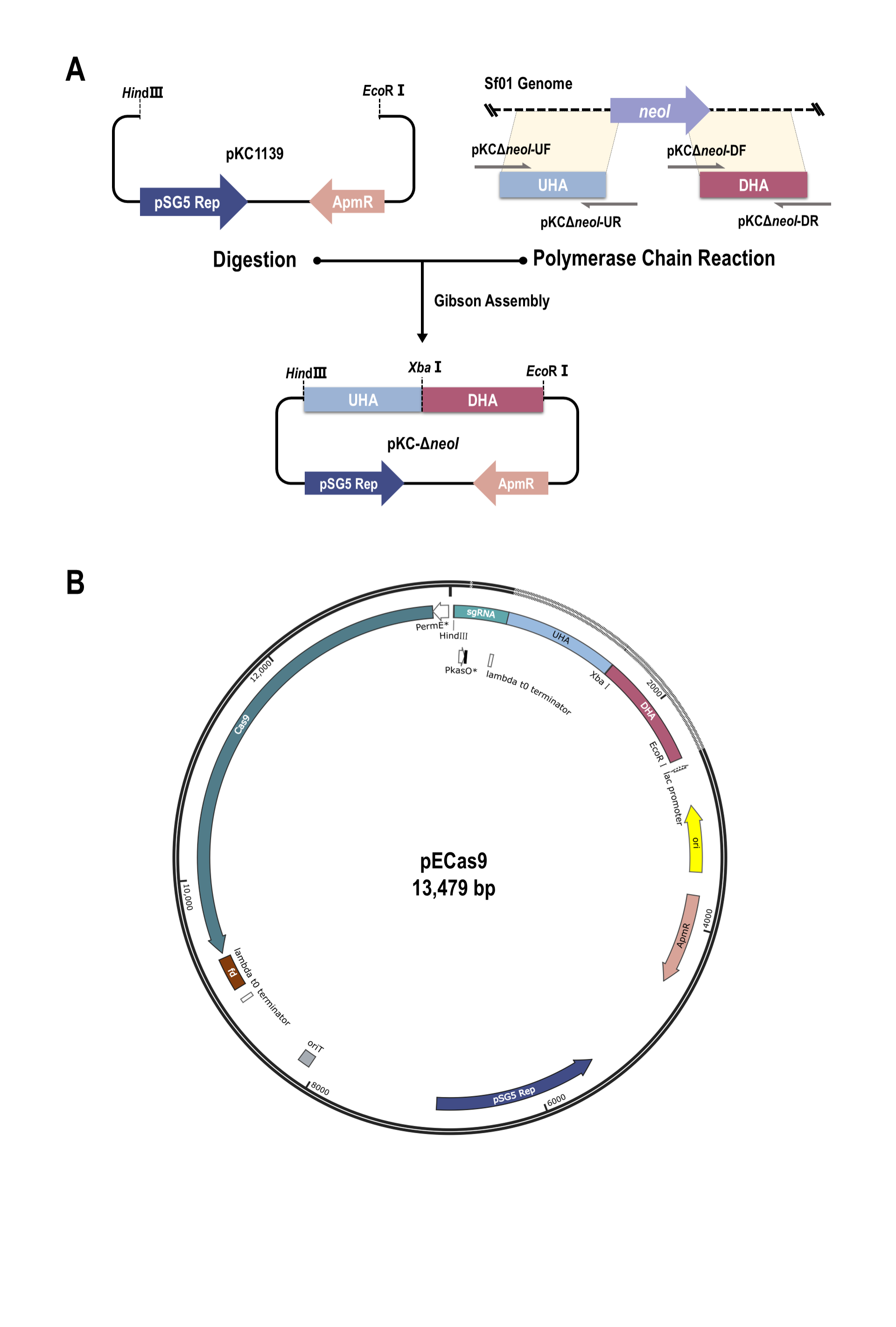

Supplement: Fig. S2 — Construction of pKC-ΔneoI, and pECas9 plasmid map. [file aem.01953-24-s0002.tiff]

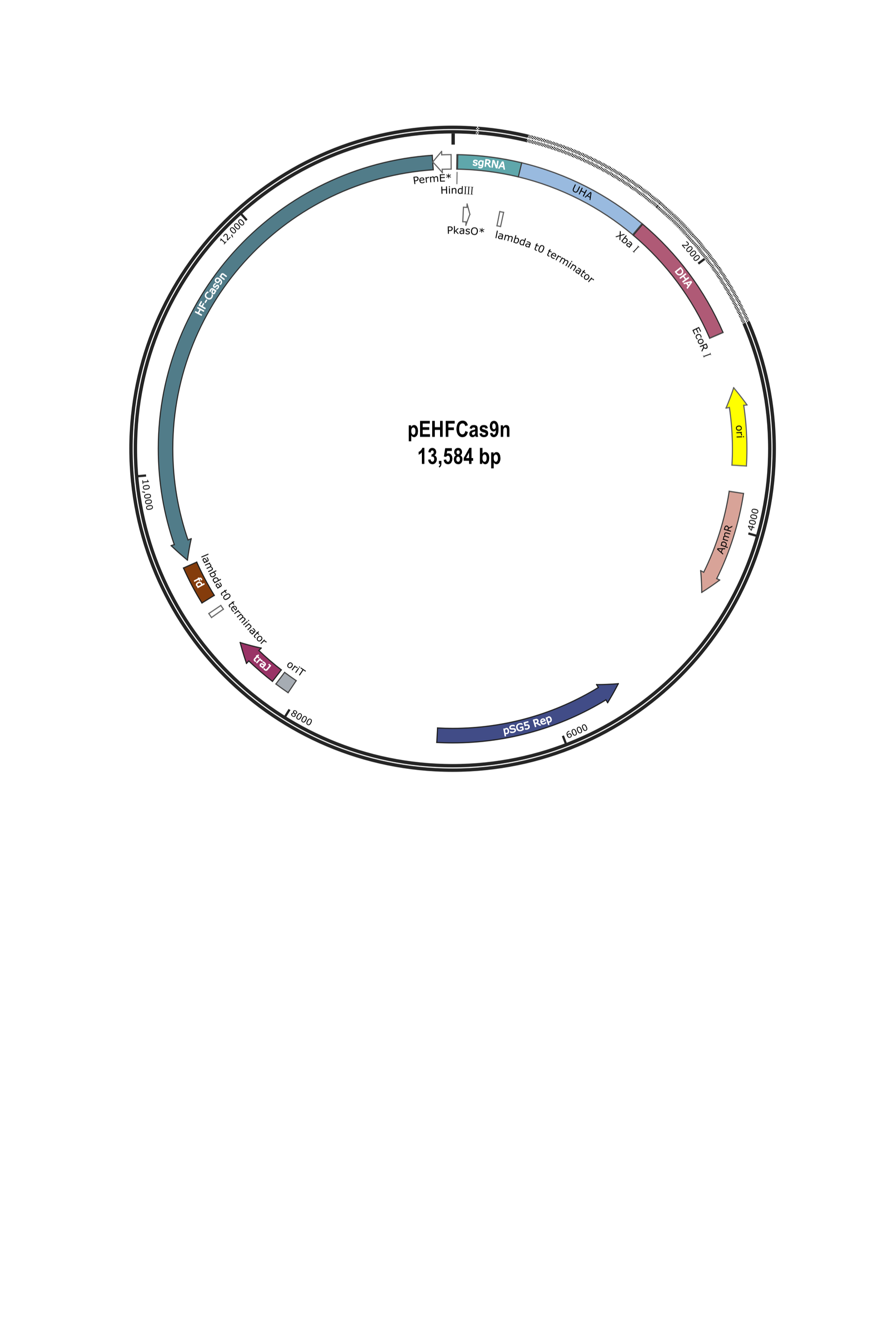

Supplement: Fig. S3 — pEHF-Cas9n plasmid map. [file aem.01953-24-s0003.tiff]

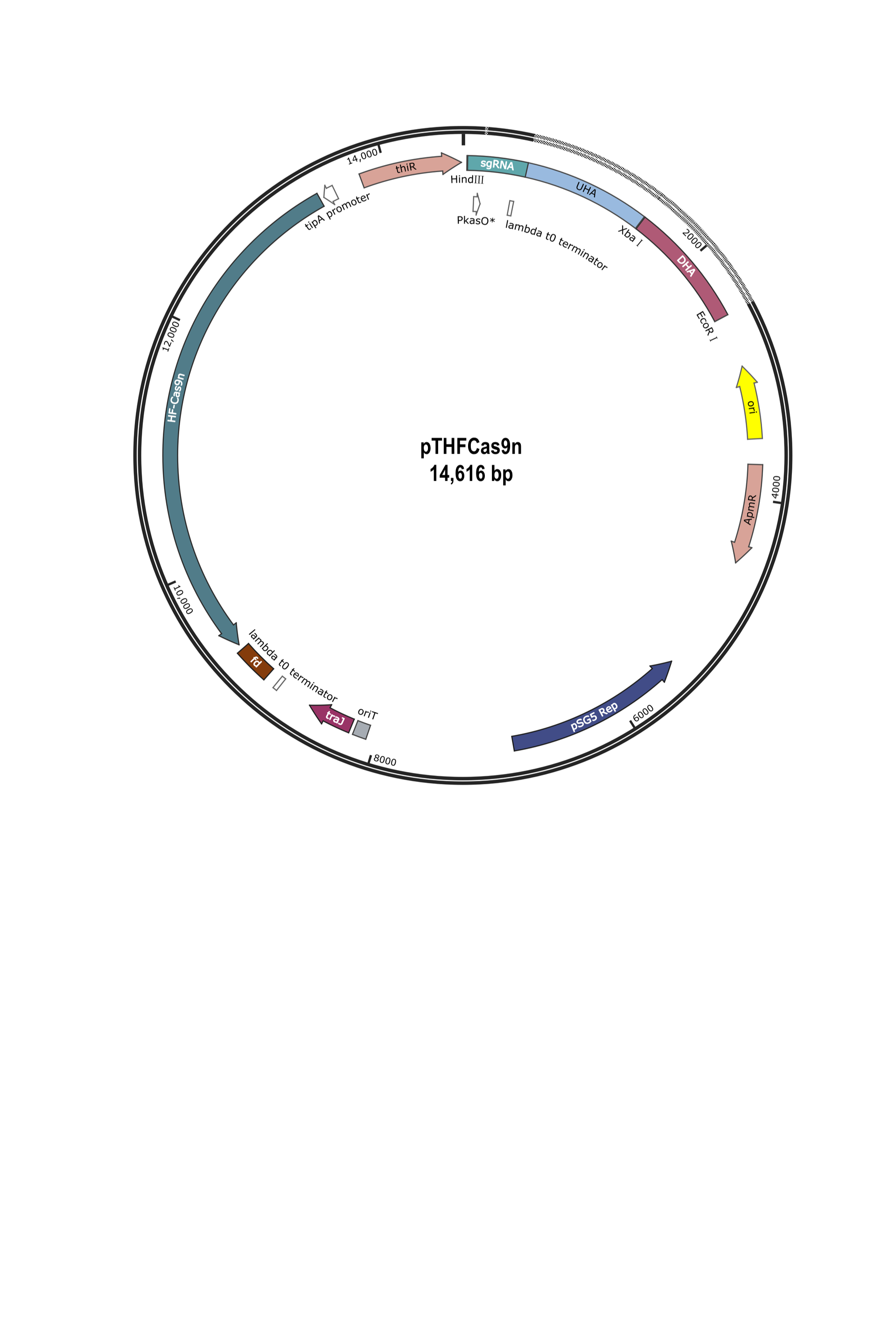

Supplement: Fig. S4 — pTHF-Cas9n plasmid map. [file aem.01953-24-s0004.tiff]

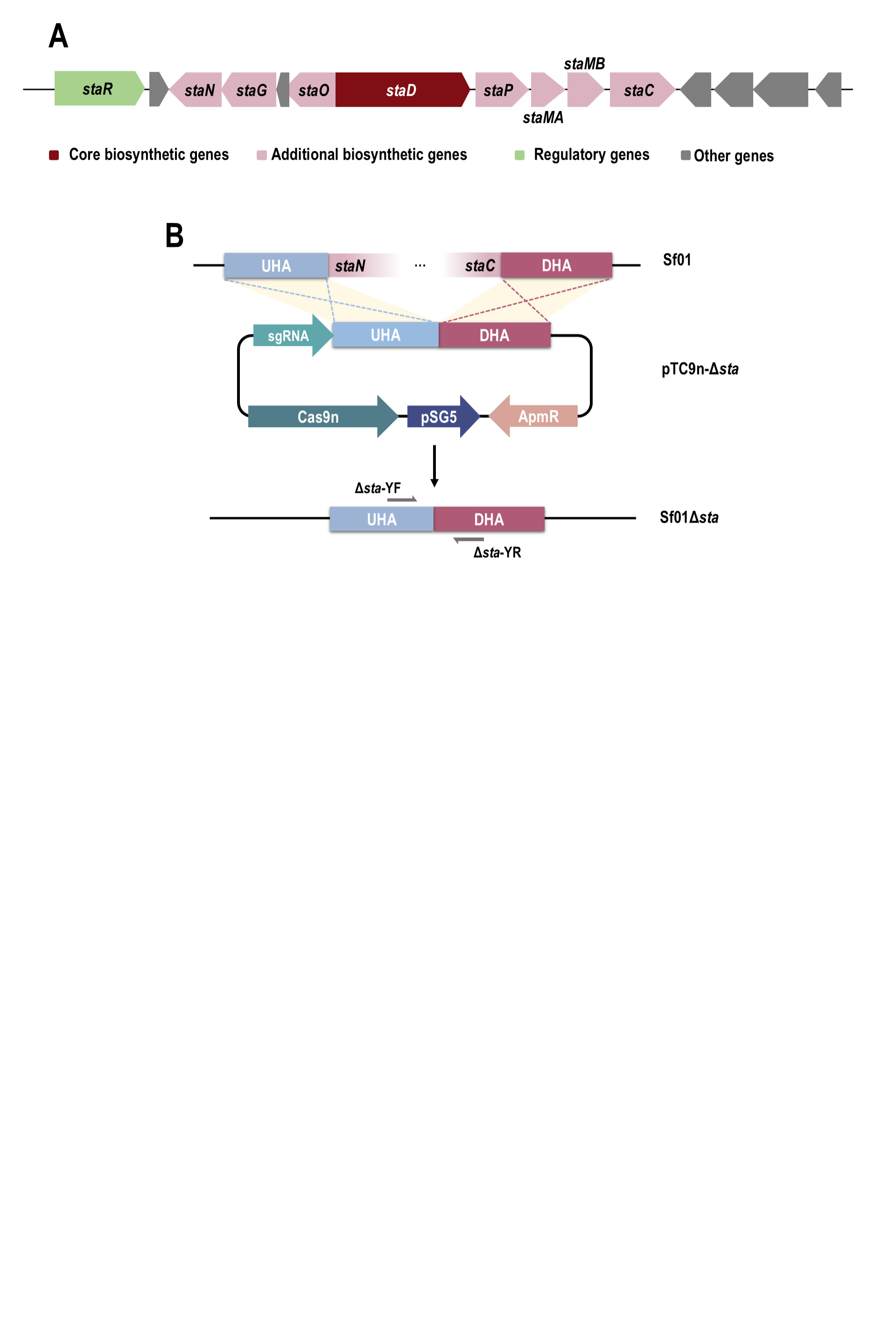

Supplement: Fig. S5 — Construction of Sf01Δsta. [file aem.01953-24-s0005.tiff]
