# Supplementary material for: Optimizing genome editing efficiency in Streptomyces fradiae via a CRISPR/Cas9n-mediated editing system
Source: Appl Environ Microbiol. 2025 Jan 22;91(2):e01953-24. doi: 10.1128/aem.01953-24 (PMC11837490; doi:10.1128/aem.01953-24)
Supplement: Supplemental material — Table S1; legends for Fig. S1 to S5. [file aem.01953-24-s0006.doc]

Supporting information

**Optimizing Genome Editing Efficiency in Streptomyces fradiae via a CRISPR/Cas9n-Mediated editing system**

**Yuhan Wu ^a#^, Hui Jin ^a#^, Qiang Yu ^a^, Zihan Wei ^a^, Jiang Zhu ^a^, Xiangqi Qiu ^b^, Junhui Li ^b^,** **Gan Luo ^b^, Yangyang Zhan^a^, Dongbo Cai ^a*^, Shouwen Chen ^a*^**

^a^ *State Key Laboratory of Biocatalysis and Enzyme Engineering*, *Environmental Microbial Technology Center of Hubei Province, College of Life Sciences, Hubei University, Wuhan, 430062, PR China*

^b^ *Lifecome Biochemistry Co. Ltd, Nanping, 353400, PR China*

**Corresponding author*: Prof. Shouwen Chen and Prof. Dongbo Cai

*Tel./fax.*: +86 027-88666081.

*E-mail address:* [mel212@126.com](mailto:mel212@126.com) (S. Chen) and [caidongbo@hubu.edu.cn](mailto:caidongbo@hubu.edu.cn) (D. Cai)

*Postal address*: 368 Youyi Avenue, Wuchang District, Wuhan 430062, Hubei, PR China

**Table S1 Calculation of mutation energy, based on PDB accession 4OO8 and 4UN3**


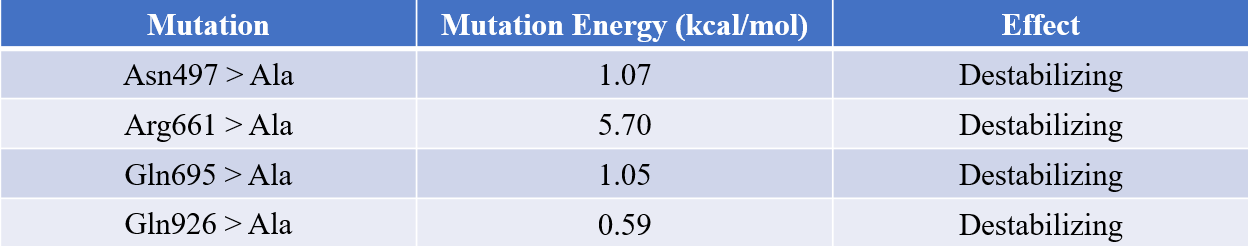


**Figure** **captions**

**Fig. S1 Optimization of conjugant parameters for *S. fradiae* Sf01. A:** Growth status of Sf01 in different solid mediums. **B:** Growth status of Sf01 in SFM solid medium containing different MgCl₂ **C:** Effect of heating temperature on conjugation efficiency. **D:** Effect of apramycin addition time on conjugation. **E:** Ratio of donor and receptor on conjugation frequency.

**Fig. S2 A:** Construction of pKC-Δ*neoI*. **B:** pECas9 plasmid map.

**Fig. S3 pEHF-Cas9n plasmid map**

**Fig. S4 pTHF-Cas9n plasmid map**

**Fig. S5 Construction of Sf01Δ*sta*. A:** Staurosporine biosynthetic gene cluster map in Sf01. **B:** Sf01Δ*sta* editing process
